# The Exodus of Young Doctors in Tanzania: Implications and Urgent Solutions

**DOI:** 10.1002/puh2.70093

**Published:** 2025-09-23

**Authors:** Majani Edward

**Affiliations:** ^1^ Department of Public Health St. Francis University College of Health and Allied Sciences Ifakara Tanzania; ^2^ Youth Health Action Network Dodoma Tanzania

**Keywords:** exodus, Tanzania, young doctors

## Abstract

The healthcare system in Tanzania is facing an alarming crisis as a recent report by researchers from the University of California, San Francisco (UCSF), and Muhimbili University of Health and Allied Sciences (MUHAS) predicts that half of the country's young doctors will leave the public healthcare system by 2025. This article explores the systemic challenges driving this exodus, including inadequate salaries, poor working conditions, limited career advancement opportunities, and persistent burnout. It also examines the global migration of young doctors in search of better opportunities and highlights gaps in medical education and mentorship as contributing factors to dissatisfaction. The implications of this attrition are profound, posing significant risks to healthcare delivery, particularly in rural and underserved areas. Workforce shortages could compromise Tanzania's progress toward universal health coverage (UHC), exacerbate inequities in healthcare access, and lead to economic losses tied to training doctors who exit prematurely. These challenges are not unique to Tanzania, as similar trends are observed across low‐ and middle‐income countries (LMICs), underscoring the global nature of healthcare workforce shortages. To address this issue, the article advocates for urgent policy reforms to improve working conditions, competitive salaries, and incentives for retention. It also calls for mentorship programs, continuous professional development, investments in public health systems, and strengthened collaborations between policymakers, healthcare institutions, and global health organizations. This article emphasizes the need for swift and sustainable solutions to safeguard the future of Tanzania's healthcare system, retain young doctors, and ensure the delivery of quality care to all citizens. The findings highlight the critical importance of prioritizing healthcare workforce retention to address both national and global health challenges.

## 1 Introduction

Young doctors represent the backbone of any healthcare system, bringing fresh energy, innovative ideas, and critical skills necessary to sustain and advance medical services [1]. They play a pivotal role in bridging healthcare gaps, especially in resource‐limited settings, where the need for accessible and quality healthcare is dire. Their contributions not only enhance service delivery but also drive public health initiatives aimed at achieving universal health coverage (UHC). However, the healthcare sector in Tanzania is facing an unprecedented challenge that threatens to erode this essential workforce [2]. According to an alarming report by researchers from the University of California, San Francisco (UCSF), and Muhimbili University of Health and Allied Sciences (MUHAS), half of young doctors are projected to leave the public healthcare system by 2025 [3]. The report suggests that young doctors who quit will not be absorbed into the public health system, creating a potential void in the workforce. The reasons for this trend are multifaceted and deeply rooted in systemic challenges such as poor remuneration, burnout, limited career progression, and unfavorable working conditions [4]. Additionally, the global demand for healthcare professionals has fueled a migration trend, as many young doctors seek better opportunities abroad. This “brain drain” not only deprives the country of its skilled workforce but also exacerbates existing health inequities [5]. Given that Tanzania is already grappling with a shortage of healthcare workers, this projected exodus could have devastating effects on the delivery of healthcare services, particularly in rural and underserved areas where access to medical professionals is already limited [6].

This article aims to delve into the implications of these findings and highlight the urgent need for policy and systemic reforms to address the underlying causes of young doctors’ dissatisfaction. It seeks to spark a conversation among policymakers, healthcare institutions, and other stakeholders about the critical steps needed to retain young doctors and strengthen the resilience of Tanzania's healthcare system. Retaining these professionals is not only essential for maintaining service delivery but also for ensuring that the country's investment in medical education yields long‐term benefits. By examining the factors driving young doctors away from the public healthcare system and exploring possible interventions, this article contributes to the broader discourse on healthcare workforce sustainability in Tanzania. Addressing this issue is not only a matter of securing the future of the medical profession but also a prerequisite for building a robust and equitable healthcare system capable of meeting the needs of all Tanzanians.

### 1.1 Why Are Young Doctors Leaving?

The exodus of young doctors from the medical profession is a multifaceted issue rooted in systemic challenges that undermine their motivation and ability to remain in the healthcare workforce. Despite their vital role in providing medical care and driving public health initiatives, young doctors face significant hurdles that push them to leave the profession or seek opportunities elsewhere [7].

One of the most prominent reasons young doctors are leaving the profession is the poor working conditions prevalent in many public health facilities [1]. Low salaries remain a critical issue, with many doctors feeling that their compensation does not reflect the years of training, expertise, and responsibility they bring to their work [8]. For instance, the average annual salary for a medical officer in Tanzania is $459,646, which translates to approximately $221 per h, which often struggles to meet the rising cost of living and the burden of student loan repayments [9]. For young doctors, who often face additional financial pressures such as student loan repayment or starting families, these inadequate wages make it difficult to sustain a decent standard of living [1].

In addition to financial constraints, burnout is a pervasive problem among young doctors. Many find themselves working in understaffed facilities with heavy workloads, leading to physical and emotional exhaustion. Long hours, lack of adequate resources, and pressure to meet patient demands contribute to a high‐stress environment that is unsustainable over time. Burnout is further exacerbated by the absence of structured support systems to help young doctors navigate the challenges of their demanding roles [10].

Furthermore, limited opportunities for career advancement discourage many young doctors from staying in the profession [11]. The lack of clear pathways for professional growth, coupled with bureaucratic hurdles, often leaves them feeling stagnant and unmotivated. Young doctors who aspire to specialize or pursue leadership roles frequently encounter barriers such as insufficient funding for postgraduate education or a lack of available slots in specialty training programs [11]. This stagnation can lead to frustration, prompting some to abandon their careers altogether or seek opportunities in fields outside of medicine.

Public health facilities, which serve as the backbone of healthcare delivery in Tanzania, have also struggled to capture and retain young talent. In many cases, these facilities lack the infrastructure, resources, and management systems needed to create an enabling environment for young doctors. Poor working conditions, coupled with the perception of neglect and underappreciation, erode the morale of young healthcare professionals [12, 13].

The “brain drain” phenomenon is another significant factor contributing to the loss of young doctors. Tanzania, like many other low‐ and middle‐income countries (LMICs), faces the challenge of retaining its healthcare workforce in the face of global demand for skilled professionals. Many young doctors, frustrated by local challenges, are drawn to better opportunities abroad, where they can benefit from higher salaries, better working conditions, and access to advanced training programs [14].

This migration trend is not only a reflection of individual aspirations but also a symptom of systemic failures. For young doctors, the decision to leave often stems from a combination of dissatisfaction with their current circumstances and the allure of a more supportive environment in high‐income countries [4]. The loss of these professionals is a significant setback for Tanzania's healthcare system, which is already struggling with a shortage of skilled workers [7].

The migration of young doctors has broader implications beyond the immediate loss of talent. It creates a vicious cycle in which the departure of skilled professionals places additional strain on those who remain, further exacerbating burnout and dissatisfaction. Moreover, the resources invested in training these doctors yield limited returns for the country, as their skills and expertise are utilized elsewhere.

Another critical issue contributing to the exodus of young doctors is the gap in medical education and mentorship. Although Tanzania has made strides in increasing the number of medical graduates, many young doctors enter the workforce feeling ill‐prepared to meet the demands of clinical practice. Inadequate training facilities, outdated curricula, and a lack of hands‐on experience during medical school leave many graduates struggling to adapt to the realities of healthcare delivery [4].

Additionally, the absence of robust mentorship programs further compounds the challenges faced by young doctors. Mentorship plays a crucial role in guiding young professionals through the early stages of their careers, providing them with the support and guidance needed to navigate the complexities of the medical profession. However, many young doctors in Tanzania report a lack of access to mentors who can help them develop their skills, set career goals, and address the challenges they encounter in their work environments [15].

Placement challenges also contribute to dissatisfaction among young doctors. In many cases, graduates are posted to remote or underserved areas with limited resources and infrastructure. Although these placements are critical for addressing healthcare disparities, they often leave young doctors feeling isolated and unsupported. The lack of opportunities for professional growth in these settings, combined with the logistical and personal challenges of working in remote locations, discourages many from continuing in their roles [12].

### 1.2 Implications for Tanzania's Healthcare System

The departure of young doctors poses a significant risk to Tanzania's healthcare system, particularly in underserved and rural areas where access to medical professionals is already limited [7]. A reduced workforce exacerbates the challenge of delivering essential health services to communities in need, leaving gaps in care and increasing the burden on the remaining healthcare providers. This attrition threatens to derail the country's progress toward achieving UHC, a critical global health objective aimed at ensuring that all individuals have access to quality healthcare without financial hardship [16].

Furthermore, a declining number of young doctors in the workforce compromises the ability to respond to public health crises and emerging health threats. Epidemics, natural disasters, and other emergencies require a robust and well‐distributed healthcare workforce. Without sufficient numbers of young doctors, the country risks being unprepared to manage these challenges effectively, jeopardizing the health and well‐being of its population [1].

The financial implications of losing young doctors are profound. Training a medical professional is a costly investment, both for the government and the individual. When these trained professionals leave the system prematurely, the returns on this investment are lost, representing a significant economic burden. The migration of doctors to other sectors or countries also contributes to a loss of intellectual capital, which is difficult to quantify but has far‐reaching consequences for healthcare innovation and development [11].

Additionally, the cost of addressing the resulting workforce shortages—through the recruitment of foreign‐trained doctors or other temporary measures—can strain limited healthcare budgets. This financial strain diverts resources that could otherwise be used to improve healthcare infrastructure, procure medical supplies, or expand public health programs [6].

The challenges facing Tanzania are not unique; many LMICs are grappling with similar issues related to healthcare workforce retention. The global shortage of healthcare workers, estimated by the World Health Organization to reach 10 million by 2030, highlights the universal nature of this problem. LMICs are particularly vulnerable as they face competition from high‐income countries that offer better salaries and working conditions [6].

Drawing parallels with other countries emphasizes the need for global cooperation in addressing workforce attrition. Sharing lessons learned and adopting best practices from countries that have successfully implemented retention strategies can provide valuable insights for Tanzania. The recognition of healthcare workforce challenges as a global issue underscores the importance of collective action in finding sustainable solutions.

### 1.3 What Can Be Done?

The combination of workforce challenges, global migration, and gaps in education and mentorship has created a perfect storm that threatens the sustainability of Tanzania's healthcare workforce. Addressing these issues requires a multifaceted approach that includes improving working conditions, offering competitive salaries, and providing clear pathways for career advancement [7]. Equally important is the need to invest in medical education and mentorship programs that equip young doctors with the skills and support they need to succeed.

Policymakers, healthcare institutions, and other stakeholders must prioritize these reforms to ensure that young doctors feel valued and supported in their roles. Retaining this critical workforce is essential for maintaining the quality and accessibility of healthcare services in Tanzania. Failure to act will not only compromise the well‐being of young doctors but also jeopardize the health of the nation as a whole.

One of the most immediate steps to address this issue is the implementation of comprehensive policy reforms aimed at improving the working conditions of young doctors. Competitive salaries, performance‐based incentives, and housing or transportation allowances can help reduce the financial strain on healthcare workers. Policies that ensure manageable workloads and promote work‐life balance are equally important in combating burnout and retaining talent [1].

Mentorship programs and opportunities for continuous professional development (CPD) are critical in supporting young doctors as they transition into their careers. A structured mentorship system can provide guidance, foster professional growth, and help doctors navigate the challenges they face in the early stages of their careers. CPD programs, on the other hand, can ensure that doctors remain up‐to‐date with advancements in medical knowledge and technology, making them feel valued and empowered [17].

Additionally, creating clear pathways for career advancement within the public health system can motivate young doctors to remain in their roles. Opportunities to specialize, participate in research, or assume leadership positions can give young doctors a sense of purpose and long‐term professional fulfillment [1].

Investing in public health systems is essential for creating an enabling environment that attracts and retains medical professionals. This includes upgrading healthcare infrastructure, ensuring the availability of medical supplies, and fostering a culture of professionalism and respect within healthcare institutions. Equally important is addressing the inequities faced by doctors working in rural or underserved areas. Providing additional support, such as hardship allowances or better living conditions, can make these postings more appealing [13].

The retention of young doctors is a complex issue that requires collaboration among policymakers, healthcare institutions, professional associations, and global health organizations. Governments must work closely with stakeholders to develop and implement evidence‐based strategies for workforce retention. Partnerships with international organizations can also provide technical and financial support as well as facilitate knowledge sharing and capacity building [6].

Moreover, engaging young doctors in the decision‐making process ensures that their perspectives and needs are taken into account. By fostering a sense of ownership and involvement, such collaboration can help build trust and commitment among healthcare workers [11].

## 2 Conclusion

The retention of young doctors represents a critical imperative with far‐reaching consequences for the sustainability and effectiveness of Tanzania's healthcare system. Without sustained and evidence‐based interventions, the loss of this vital workforce has the potential to undermine progress toward UHC, compromise the quality of healthcare delivery, and place undue strain on the nation's healthcare infrastructure. The findings of the UCSF and MUHAS report underscore the urgency for all stakeholders involved in Tanzania's healthcare sector to prioritize this issue. Focused reforms are necessary to improve working conditions, expand opportunities for professional growth, and address the systemic challenges that contribute to young doctors leaving the public sector. Strengthening mentorship programs, strategically investing in public health facilities, and fostering robust collaboration across sectors are essential steps toward building a resilient and sustainable healthcare workforce. By implementing targeted and evidence‐informed solutions, Tanzania can work toward safeguarding its healthcare future, ensuring the well‐being of its population, and potentially serving as a model in addressing a significant global health challenge.

## Author Contributions

**Majani Edward**: conceptualization, writing – original draft, writing – review and editing, supervision, investigation, methodology, formal analysis.

## Ethics Statement

With respect to this type of article, we did not seek informed consent; we used existing data from the prior findings.

## Consent

Data were collected from the prior findings so no informed consent was employed.

## Conflicts of Interest

The author declares no conflicts of interest.

## Data Availability

Data were collected from the prior findings.
